# Meningococcal Carriage in Children with Atypical Hemolytic Uremic Syndrome Receiving Eculizumab Therapy

**DOI:** 10.3390/children11101164

**Published:** 2024-09-25

**Authors:** Asli Kavaz Tufan, Fatma Ozak Batibay, Gulsah Kaya Aksoy, Bora Gulhan, Beltinge Demircioglu Kilic, Ismail Dursun, Bahar Buyukkaragoz, Aysun Caltik Yilmaz, Hulya Nalcacioglu, Tulay Becerir, Nuran Cetin, Kubra Celegen, Meltem Dinleyici, Mucahit Kaya, Omer Kilic, Ener Cagri Dinleyici

**Affiliations:** 1 Department of Pediatric Nephrology, Faculty of Medicine, Eskisehir Osmangazi University, 26040 Eskisehir, Türkiye; aslikavaz@ogu.edu.tr (A.K.T.); fatma.ozakbatibay@ogu.edu.tr (F.O.B.); nurancetin@ogu.edu.tr (N.C.); 2 Department of Pediatric Nephrology, Faculty of Medicine, Akdeniz University, 07070 Antalya, Türkiye; gulsahaksoy@akdeniz.edu.tr; 3 Department of Pediatric Nephrology, Faculty of Medicine, Hacettepe University, 06230 Ankara, Türkiye; bora.gulhan@hacettepe.edu.tr; 4 Department of Pediatric Nephrology, Faculty of Medicine, Gaziantep University, 27310 Gaziantep, Türkiye; beltingeiklim@gantep.edu.tr; 5 Department of Pediatric Nephrology, Faculty of Medicine, Erciyes University, 38280 Kayseri, Türkiye; idursun@erciyes.edu.tr; 6 Department of Pediatric Nephrology, Faculty of Medicine, Gazi University, 06560 Ankara, Türkiye; bbuyukkaragoz@gazi.edu.tr; 7 Department of Pediatric Nephrology, Ankara Etlik City Hospital, 06170 Ankara, Türkiye; aysundr@hotmail.com; 8 Department of Pediatric Nephrology, Faculty of Medicine, Ondokuz Mayis University, 55139 Samsun, Türkiye; hulya.nalcacioglu@omu.edu.tr; 9 Department of Pediatric Nephrology, Faculty of Medicine, Pamukkale University, 20160 Denizli, Türkiye; tbecerir@pau.edu.tr; 10 Department of Pediatric Nephrology, Kayseri Research and Training Hospital, 38050 Kayseri, Türkiye; kubractf@hotmail.com; 11 Department of Social Pediatrics, Faculty of Medicine, Eskisehir Osmangazi University, 26040 Eskisehir, Türkiye; mdinleyici@ogu.edu.tr; 12 Diagen Biotechnology, 06070 Ankara, Türkiye; m.kaya@diagen.com.tr; 13 Department of Pediatric Infectious Disease, Faculty of Medicine, Eskisehir Osmangazi University, 26040 Eskisehir, Türkiye; okilic@ogu.edu.tr; 14 Department of Pediatrics, Faculty of Medicine, Eskisehir Osmangazi University, 26040 Eskisehir, Türkiye

**Keywords:** atypical hemolytic uremic syndrome, eculizumab, *Neisseria meningitidis*, children, meningococcal vaccines, meningococci, nasopharyngeal colonization

## Abstract

Background/Objectives: Eculizumab is a first-line treatment for atypical hemolytic uremic syndrome (aHUS), and patients undergoing eculizumab therapy may become more susceptible to infection caused by *Neisseria meningitidis* (*Nm*). While meningococcal vaccination is required for patients undergoing eculizumab therapy, there is limited knowledge about meningococcal carriage in children with aHUS. We aimed to evaluate (1) the prevalence of *Nm* carriage, (2) serogroup distribution, and (3) the immunization status of children undergoing eculizumab treatment for aHUS. Methods: The Meningo-aHUS study is a prospective, multi-center study evaluating meningococcal carriage in children and adolescents in Türkiye receiving eculizumab for aHUS. We noted the age, gender, daycare, school, or university attendance, passive smoking status, previous infection and antibiotic use, and previous immunization history, including meningococcal vaccines, from the medical records of those children with aHUS. We collected nasopharyngeal samples, tested them for *Nm* using real-time polymerase chain reaction, and performed a serogroup analysis on the positive samples. Results: We collected nasopharyngeal samples from 62 children with aHUS. Out of 62 children, 61 (98.4%) had received at least one dose of the meningococcal vaccine. The median time since the last meningococcal vaccine dose was 15 months (1–59 months). We detected meningococcal carriage in three (4.8%, 95% CI 1.0–13.5) children, and all three strains were non-groupable (NG). No other serogroups were detected. Conclusions: Almost all the children received their risk-group meningococcal immunization, including booster doses. A 4.8% of children with aHUS carried NG meningococci and, no vaccine serogroups were detected. Patients treated with eculizumab remain profoundly susceptible to IMD due to these NG meningococcal strains. The occurrence of breakthrough cases and carriage of *Nm*, especially NG strains, highlights the significance of maintaining a state of constant alertness, promptly seeking medical attention, and swiftly treating any symptoms that align with IMD, regardless of their vaccination status or antibiotic prophylaxis.

## 1. Introduction

Atypical hemolytic uremic syndrome (aHUS) is a rare and potentially life-threatening disease caused by alternative complement pathway dysregulation, which leads to systemic thrombotic microangiopathy, generally characterized by hemolytic anemia, thrombocytopenia, and acute renal failure and often resulting in end-stage renal disease [1,2,3]. Although onset may occur at any age, 40% of patients develop aHUS by 18 years of age. Complement gene mutations or factor H autoantibodies have been identified in 50% to 60% of patients with aHUS [1]. Without treatment, aHUS is associated with end-stage kidney disease, disease recurrence, and mortality [1,3,4]. While aHUS was managed with plasma exchange/plasma infusion in addition to supportive treatment based on general principles of acute kidney injury until 2009, success was inconsistent [2]. Atypical HUS is usually caused by dysregulation of the alternative complement pathway, resulting in the overactivation and excessive production of the terminal attack complex, thereby leading to endothelial cell injury [1,2,3]. Eculizumab is a recombinant humanized chimeric anti-C5 monoclonal antibody, which binds to complement C5, preventing C5 cleavage and the formation of C5a and C5b-9, thus blocking the C5a pro-inflammatory and C5b-9 (terminal membrane attack complex) pro-thrombotic consequences of complement activation [5,6,7]. Eculizumab has demonstrated effectiveness and safety in pediatric patients with suspected aHUS. It is recommended as the initial treatment and should be promptly begun [1,3,8]. Administering eculizumab early results in enhancements in hematologic, kidney, and systemic symptoms in individuals with aHUS, even if they rely on dialysis. It has also been linked to enhanced quality of life and increased survival rates [1,2,7,9]. In addition to aHUS, eculizumab is licensed for the treatment of paroxysmal nocturnal hemoglobinuria (PNH), generalized myasthenia gravis, and neuromyelitis optica spectrum disorder [7,10,11,12].

The complement system is involved in innate immunity, and complement activation also prevents the formation of the membrane attack complex, which is the major defense mechanism against *Neisseria meningitidis* (*Nm*) [13,14]. Therefore, eculizumab increases the risk of invasive meningococcal disease (IMD), like in patients with congenital terminal complement component deficiency [11,14]. Eculizumab-treated patients are at an estimated 1000–2000-fold increased risk of IMD [15]. A higher increased risk of IMD has been reported in Japan, with the IMD risk in patients on eculizumab being 6100 times higher than in the normal population; furthermore, the mortality rate from IMD in patients treated with eculizumab is estimated to be 13,000 to 114,000 times that of IMD in the general population [11].

Therefore, children and adults who are started on eculizumab treatment should be immediately vaccinated against *Nm* serogroup types A, B, C, Y, and W. The vaccination protocol must incorporate the quadrivalent conjugate vaccine (MenACWY) and the serogroup B vaccine (MenB) according to established schedules. Furthermore, booster doses of the MenACWY vaccine should be given every five years, whereas MenB vaccine doses should be given every three years if eculizumab therapy continues [1,7,16,17]. It is recommended to give antibiotic prophylaxis for a minimum of two to four weeks after the immunization schedule or until protective antibody levels are confirmed. [1,7,17]. Long-term antibiotic prophylaxis for eculizumab recipients is recommended in some countries, such as the United Kingdom and France; however, this is not routine in all countries [12,18]. Neither vaccines nor antibiotic prophylaxis guarantee full protection from IMD [10]. There have been some reports of eculizumab recipients developing IMD caused by vaccine serogroups as well as disease caused by non-groupable (NG) meningococcal strains [12,15,19]. Meningococcal vaccination is not part of the national immunization program for children in Türkiye, but MenACWY-TT, MenACWY-CRM, MenACWY-D, and 4CMenB are privately available. All patients treated with eculizumab should be immunized with the tetravalent conjugate vaccine MenACWY and the MenB vaccine in accordance with their age. Re-vaccination with the tetravalent conjugate vaccine MenACWY should be performed every five years and with the MenB vaccine every two to three years [20].

*Neisseria meningitidis* is commonly carried in the human nasopharynx, especially in adolescents and young adults. Only a small fraction of carriers will develop IMD, usually shortly after acquiring the bacterium [21]. The prevalence of meningococcal carriage and serogroup distribution is critical in assessing IMD epidemiology and formulating potential immunization strategies [21,22,23]. Serogroup distribution of carriers varies according to geographical regions and changes over time in the same geographical region [24]. There is no information about meningococcal carriage in eculizumab-treated children with aHUS. The aim of this study was to evaluate (1) the prevalence of *Nm* carriage, (2) serogroup distribution, and (3) the immunization status of children receiving eculizumab due to aHUS.

## 2. Materials and Methods

### 2.1. Definition of This Study

The Meningo-aHUS study is a prospective, multi-center study evaluating meningococcal carriage among children and adolescents receiving eculizumab due to aHUS. The nine centers from seven cities that participated in this study are tertiary referral centers for pediatric nephrology. Of these nine hospitals, seven were university hospitals, and two were training and research hospitals.

### 2.2. Inclusion End Exclusion Criteria

We retrospectively reviewed the patient records of these centers and identified patients diagnosed with aHUS. We evaluated the medical records of these patients based on this study’s inclusion and exclusion criteria. aHUS were defined as a triad of Coombs-negative hemolytic anemia (hemoglobin level < 10 gr/dL, presence of schistocytes and fragmented erythrocytes in peripheral blood smear), thrombocytopenia with a platelet count < 150 × 10^9^/L, and acute kidney injury. All patients have been screened for ADAMTS-13 deficiency. The activity of ADAMTS13 above 10% was considered normal and has been included. Patients with HUS secondary to drugs, an autoimmune disease, infection (caused by Shiga-toxin-producing *Escherichia coli*, *Streptococcus pneumoniae*, and other infections), bone marrow or solid organ transplantation, or cobalamin C deficiency were excluded. We did not include patients with aHUS over 18 years old who were under follow-up care in adult nephrology clinics. The exclusion criteria included primary immune deficiencies, secondary immune deficiencies, such as human immunodeficiency syndrome, malignant hematological disorders, chronic conditions other than aHUS, and patients receiving immunosuppressive therapies other than eculizumab, whose drug treatment had ended. The evaluation of all eligible children and adolescents took place during their routine follow-up visits. We also excluded patients who did not receive medical treatment and who received information about this study but lacked parental consent.

### 2.3. Demographic, Clinical Data, and Immunization Status

We noted the children’s age, gender, daycare or school attendance, passive smoking exposure at home, previous meningococcal (MenACWY-TT, MenACWY-CRM, MenACWY-D, 4CMenB), pneumococcal (conjugated or polysaccharide vaccine), and *Haemophilus influenzae* type b vaccine history, previous antibiotic use, and upper respiratory tract infections in the last one and/or three months, as well as the previous COVID-19 infection status of the children and family members over the last year.

### 2.4. Primary and Secondary Endpoints

The primary endpoint of this study was the prevalence of *Nm* carriage and serogroup distribution (serogroups A, B, C, E, H, X, W, Y, and Z) based on age group. The secondary endpoints were the clinical characteristics of children with a positive meningococcal carriage rate and the immunization status of children with aHUS.

### 2.5. Sampling and Laboratory Analysis

Cotton swabs (Copan Diagnostics, Carlsbad, CA, USA) were used to collect nasopharyngeal samples. These samples were then placed in Amies medium with activated charcoal (DeltaLab, Barcelona, Spain). They were relocated to a laboratory equipped with charcoal Amies transport tubes. The process of extracting DNA, assessing the presence of *Nm*, and determining the serogroup in all samples that tested positive for *Nm* was carried out [25]. A single-tube, multiplex PCR assay was conducted to simultaneously detect bacterial agents. For each analysis, the resulting reaction mixture had a volume of 22 μL, which was modified to include 10 µL of DNA. A 1× PCR reaction was generated by combining 1 μL of 2.5 pmol primer with DiagenT11.1 (Diagen Biotech., Ankara, Türkiye) buffer mix in a total volume of 11 μL. The PCR analysis was conducted using the Applied Biosystems Veriti 96 Well Thermal Cycler (Waltham, MA, USA) with the following parameters: an initial denaturation cycle at 95 °C for 5 min, followed by 40 cycles at 95 °C for 1 s, 61 °C for 61 s, and 72 °C for 5 s. The final elongation step was performed at 72 °C for 5 min. SodC, CtrA, and PorA were used for general screening. Furthermore, the tauE and metA genes were utilized [26]. The products A (Orf-2), B (Sia D), C (Sia D), Y (Sia D), X (CtrA), and W (Sia D) were examined using a 2% agarose gel. Additionally, the products E (cseE), Z (cszC), and H (cshC) were also evaluated. Verification throughout the investigation was conducted using both positive and negative controls. The serogrouping study involved utilizing the *Nm* real-time PCR serogrouping kit to confirm the weak bands [24]. Verification was conducted for this purpose.

### 2.6. Statistical Analysis

We used the JASP statistical analysis application (JASP 0.16.4 version, Amsterdam, The Netherlands) to perform the statistical analyses. We presented the qualitative variables as frequencies and represented the quantitative variables by their mean value plus or minus the standard deviation if normally distributed, or by the median if not normally distributed. We used independent *t*-tests to compare continuous data that followed a normal distribution and employed Mann-Whitney U tests to evaluate data that did not follow a normal distribution. We assessed the relationships between qualitative variables using a chi-square test. We considered a *p*-value less than 0.05 as statistically significant.

## 3. Results

**Figure 1:**
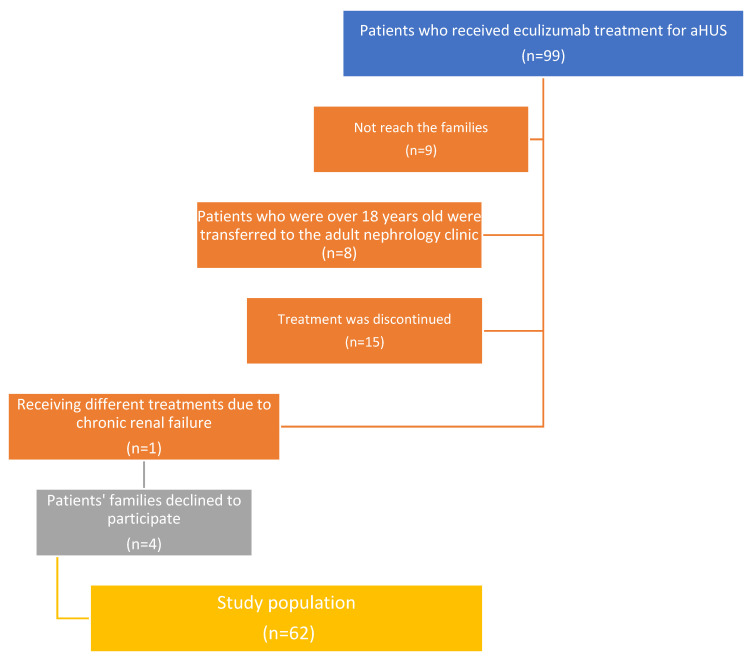
Flow chart of study population regarding exclusion reasons.

We identified 99 patients who received eculizumab treatment for aHUS. This study continued with a total of 62 patients; the reasons for exclusion have been shown in Figure 1. In this study, nasopharyngeal samples were collected from 62 children (25 girls and 37 boys, aged between 19 months and 216 months; median age 126.5 months) with aHUS undergoing eculizumab therapy. The median age of the aHUS diagnosis was 38 months (1–190 months). The median total duration of eculizumab therapy was 42.5 months. Twenty-three children (37.1%) tested positive for aHUS following genetic tests. One child had a history of meningococcemia (blood culture yielded positive for *Nm*; serogroup definition was not performed). A total of 35 children (56.5%) attended a daycare center or school; 37 children (59.7%) had indoor smoking exposure; and 1 child was an active smoker. The median number of household members was five, with a minimum of three and a maximum of eight. Eighteen children (29%) had had an upper respiratory tract infection (URTI) in the last month. Seventeen children (27.4%) had used antibiotics in the last month, and twenty-seven children (43.5%) had used antibiotics in the last three months. None of the patients or their household members had a PCR-positive COVID-19 infection in the past year (Table 1).

Immunization status: 61 out of 62 children (98.4%) had received at least one dose of the meningococcal vaccines; 23 children (37.0%) had received the MenACWY-D and 4CMenB vaccines; 32 children (51.6%) had received the MenACWY-TT and 4CMenB vaccines; and 2 children (3%) had received the MenACWY-CRM and 4CMenB vaccines. Three children had only received the MenACWY-D vaccine, and one child had only received the MenACWY-TT vaccine. One child was completely unvaccinated due to previous allergic reactions to vaccine components. The median time lapse since the last meningococcal vaccine dose was 15 months (1–59 months). All children had received age-appropriate conjugated pneumococcal vaccines (the 13-valent conjugated pneumococcal vaccine, PCV13), and 45 children had received the additional 23-valent polysaccharide pneumococcal vaccine (PPSV23). Sixty-one children had received the age-appropriate *Haemophilus influenzae* type B vaccine and received a booster dose. Only two children had received antibiotic (amoxicillin clavulanic acid) prophylaxis. One of the patients had prolonged antibiotic prophylaxis because of a previous history of meningococcemia. The other patient could not receive any vaccine because of a previous history of anaphylaxis.

Meningococcal carriage was detected in three (4.8%, 95% CI 1.0–13.5) of the participants through an rt-PCR test. A serogroup distribution of the three *Nm* strains isolated from the nasopharyngeal specimens was revealed as non-groupable strains. No serogroups A/B/C/W/Y/X/E/H/Z were detected. There are no statistical differences between carriers and non-carriers regarding demographic, clinical, and risk factors (Table 1).

### Characteristics of Carrier Patients with aHUS

Case 1: A 19-month-old boy was diagnosed with aHUS at 11 months of age and had been receiving eculizumab for the last 8 months. He was administered two doses each of the MenACWY-TT and 4cMenB vaccines. He did not attend a daycare center and lived with three people, none of whom smoked. He also received antibiotic prophylaxis and had no URTI or antibiotic use in the last month.

Case 2: A 106-month-old girl was diagnosed with aHUS at 47 months of age and had been receiving eculizumab for the last 47 months. She was administered one dose of MenACWY-TT and two doses of 4cMenB. She attended school, lived with four people, and was exposed to passive smoking. She did not receive antibiotic prophylaxis and used antibiotics in the last month.

Case 3: A 140-month-old girl was diagnosed with aHUS at 40 months of age and had been receiving eculizumab over the last 100 months. She was administered one dose of MenACWY-TT and two doses of 4cMenB and had received booster doses 27 months ago. She attended school, and there were four people in the home. She did not receive antibiotic prophylaxis, and neither did she have a URTI or use antibiotics in the last month.

## 4. Discussion

In this study, we first showed the meningococcal carriage rate and serogroup distribution in children diagnosed with aHUS. Approximately all the children had received the meningococcal vaccine. In our series, two children with aHUS received antibiotic prophylaxis due to a history of meningococcemia and a previous anaphylactic reaction to a vaccine component. The meningococcal carriage rate was 4.8% in children with aHUS, all NG strains, and no vaccine serogroups were detected. While non-groupable strains are often found in the nasopharynx with no symptoms and do not usually cause IMD in healthy people, eculizumab-treated patients are highly likely to contract IMD because of NG meningococcal strains [12,15,19]. It is not known whether the MenACWY and 4CMenB vaccines provide sufficient cross-protection against NG strains [18].

Although no previous studies have shown non-groupable meningococcal carriage in patients with aHUS, IMD associated with non-groupable strains has been reported. The Centers for Disease Control and Prevention reported that between 2008 and 2016, there were 16 cases of IMD (aged between 16 and 83 years, 5 with aHUS) among patients using eculizumab, with NG strains causing the most cases (11 of 16 patients) [15]. Socie et al. [9] reported 76 cases of IMD (8 under 16 years old) between 2007 and 2016, with the most common serotypes being NG and B. Ladhani et al. [27] reported 16 cases of IMD between 2008 and 2017 in patients with complement deficiencies. Of the nine patients who received eculizumab therapy (six with PNH and three with aHUS), three isolates were caused by NG but were genetically in capsular group B. The occurrence of IMD in individuals with hereditary complement deficiencies was caused by encapsulated strains. However, in patients undergoing eculizumab therapy, four of the nine IMD episodes were caused by either the NG or group E strains. These strains have lower virulence and are typically only linked to carriage [27]. There have been documented cases of deaths caused by the NG strains in eculizumab-administered patients. Nolfi-Donegan et al. [19] reported fatal meningococcemia caused by NG strain in a fully vaccinated 16-year-old girl with PNH only 24 h after the second eculizumab dose. They showed that neither the MenB vaccination, which matched two antigens (the factor H binding protein and the Neisseria heparin binding antigen), nor the high serum antibody titers prevented the rapidly fatal disease due to NG [19]. Several case reports and case studies involving both children and adults have documented instances of patients receiving eculizumab treatment and subsequently developing meningococcemia, despite having received immunizations or a combination of chemoprophylaxis [18,19,28,29,30,31,32,33,34,35]. Studies have reported that the response to vaccine serogroups may vary in patients receiving eculizumab [28,36,37]. Additionally, antibodies developed against vaccine serotypes do not provide cross-protection against NG serotypes [38,39]. Fortunately, in our study, vaccine serogroups were not detected as carriers.

In Türkiye, meningococcal disease and carriage seroepidemiology in healthy children are dynamic and differ significantly from those in other countries [40]. Serogroup B dominates the meningococcal seroepidemiology of IMD, followed by serogroups W, A, and Y, with serogroup C remaining undetected in children since 2005 [41,42]. In 2023, we conducted a study on meningococcal carriage in children and young adults aged between 0 and 24 years, detecting meningococcal carriage in 8.4% of the participants, with NG making up 45.5%, serogroup B making up 30.5% [43]. In the present study, NG meningococcal carriage in eculizumab-treated children with aHUS was like the results reported in our 2023 study. We did not detect other serogroups, which could be related to the potential vaccine effects on vaccine serogroup carriage. MenACWY vaccines have been shown to lower pharyngeal meningococcal carriage [44]. Lebel et al. [45], suggested an approach that includes immunizing the close household contacts of patients being treated with eculizumab and regularly checking to see whether any of these contacts are *Nm* carriers so that they can be given a targeted, susceptibility-based antibiotic course.

We have some limitations. In this study, we did not perform culture for the detection of *N. meningitidis*, so we were not able to perform more detailed molecular analyses and antibiotic resistance evaluations for the strains. This was not an obstacle for meningococcal carriage frequency and serogroup determination because we evaluated more than one gene region in the PCR method we used in our study. This study only took one patient swab sample, but taking samples at different follow-up times would have been helpful. Since the distribution of serogroups for meningococcal carriage may vary between countries, it may be useful to conduct studies in other countries. Some of the patients used antibiotics in the last 1–3 months, which may have affected our results. However, our study’s strengths were the number of patients with this rare disease, the test characteristics we used, and the fact that it was the first carrier study.

Vaccination against A, C, W, Y, and B serotypes is a crucial step in disease control, especially in high-risk groups, such as patients receiving eculizumab [9]. Moreover, a long-term or lifelong course of eculizumab treatment is expected for some patients, and routine antibiotic prophylaxis, antibiotic selection, dose and dose interval, and patient compliance remain controversial. Licensed indications for eculizumab use have expanded, and many additional complement inhibitors, including ravulizumab, crovalimab, avacopan, nomacopan, iptacopan, and cemdisiran, have been developed or are under consideration [2]. This suggests that the number of people with an elevated risk for IMD due to complement inhibitor use might increase. Therefore, it is essential to assess the effectiveness of existing and new strategies for preventing meningococcal disease, which is crucial for this population.

## 5. Conclusions

In this study, 4.8% of children with aHUS carried NG meningococci, and no vaccine serogroups were detected. Patients treated with eculizumab remain profoundly susceptible to IMD due to these NG meningococcal strains. The occurrence of breakthrough cases and carriage of *Nm*, especially NG strains, underscore the need for healthcare providers and patients to have a high index of suspicion for IMD and early appropriate treatment for any symptoms that align with IMD, regardless of their vaccination status or antibiotic prophylaxis. The Meningo-aHUS study is the first pivotal study in this patient group, and in the second part of this study, we plan to evaluate meningococcal carriage, serogroup distribution, and vaccine response in a larger series of patients with aHUS.

## Figures and Tables

**Table 1:** Demographical, clinical factors, and risk factors related to meningococcal infections in total, carrier, and non-carrier study groups.

	*Nm* Carriers n = 3	Non-Carriers n = 59	Total n = 62
Gender (girls/boys)	2/1	23/36	25/37
Age (months)	106 (19–140)	129 (23–216)	126.5 (19–216)
Age of the aHUS diagnosis (months)	40 (11–47)	38 (1–190)	38 (1–190)
Total duration of eculizumab therapy (months)	59 (8–100)	41 (2–180)	42.5 (2–180)
Meningococcal vaccination	3/3	58/59	61/62
Time-lapse since the last meningococcal vaccine dose	17 (5–52)	14.5 (1–59)	15 (1–59)
Antibiotic prophylaxis	0/3	2/59	2/62
Daycare/school attendance	2/3	33/59	35/62
Upper respiratory tract infection history (last month)	0/3	18/59	18/62
Antibiotic use history (last month)	0/3	17/59	17/62
Previous COVID-19 infection	0/3	0/59	0/62
Previous COVID-19 infection history among householders	0/3	0/59	0/62
Smoking at home	1/3	36/59	37/62
Number of household members	4 (3–4)	5 (3–8)	5 (3–8)

*Nm*: *Neisseria meningitidis*.

## Data Availability

The data are available from the corresponding author upon reasonable request. Data is not available publicly due to technical and ethical reasons.
